# Neurological Complications Associated With the Powassan Virus and Treatment Interventions

**DOI:** 10.7759/cureus.71780

**Published:** 2024-10-18

**Authors:** Chinemerem Ndukwe, Allison C Melville, Mubarak Osman, Yakubu Mohammed, Melvin Oduro, Paa Kwesi Ankrah

**Affiliations:** 1 Biology, Georgia State University, Atlanta, USA; 2 Ambulatory Surgery Center, Duke University Health System, Durham, USA; 3 Medicinal Chemistry, East Tennessee State University, Johnson City, USA; 4 Chemistry, Texas A&amp;M University–Commerce, Commerce, USA; 5 Infectious Diseases, Duke University, Durham, USA

**Keywords:** antiviral therapy, encephalitis, meningitis, neurological disorders, powassan virus, tick-borne diseases

## Abstract

Powassan virus (POWV), a flavivirus transmitted by *Ixodes* ticks, poses significant neurological risks, including encephalitis and meningitis. Despite its growing geographical spread and severe clinical manifestations, no specific antiviral treatments are currently available. This review synthesizes the existing literature on POWV, focusing on its epidemiology, clinical manifestations, neurological impact, and current treatment approaches. The review also explores the molecular biology of POWV, highlighting its genomic structure and the role of nonstructural proteins in its pathogenesis. POWV infection can range from asymptomatic to severe neurological disorders such as encephalitis and meningitis, with the latter presenting less severe outcomes. The pathophysiology of the virus involves immune evasion and robust neuroinflammation, complicating treatment strategies. Current management is supportive, with efforts ongoing to repurpose existing antivirals and develop new therapeutic interventions. Prognostic disparities between POWV-induced meningitis and encephalitis are notable, with meningitis generally having more favorable outcomes. The review also identifies critical gaps in POWV research, particularly in understanding the long-term neurological impacts and developing specific antiviral therapies. This article collates existing relevant literature on the POWV and culminates in two strategic actions: firstly, in the absence of an existing vaccine, it proposes repurposing antiviral drugs designed for related flaviviruses to target shared molecular pathways, such as nonstructural proteins in the POWV. Secondly, the article advocates for longitudinal studies to explore the long-term neurological effects on individuals who have survived POWV infections.

## Introduction and background

This review presents a comprehensive examination of the Powassan virus (POWV), elucidating its epidemiology, clinical manifestations, and therapeutic challenges, with particular emphasis on neurological complications. Initially identified in Powassan, Canada, in 1958, POWV has since demonstrated a broader geographic distribution. The virus is categorized into two lineages: POWV lineage I, predominant in North America, and lineage II, more prevalent in Russia and parts of Europe [1]. Clinical presentations of POWV infection exhibit a spectrum ranging from asymptomatic cases to severe neurological complications. The mortality rate for POWV is 15% upon neuroinvasion of the virus [2]. Non-neuroinvasive infections often present in asymptomatic individuals and appear healthy. There are currently no FDA-approved drugs or vaccines for POWV infections, with a characteristic rash emerging one to two weeks post-infection [1,2]. Current research underscores the critical role of host immune response in disease progression, with factors such as viral load, strain genotype, host age, immune status, and co-infections significantly influencing the severity of clinical outcomes [2].

Nemeth et al. delineate the natural transmission cycle of POWV, which involves small mammals, primarily rodents and squirrels, serving as reservoir hosts [3]. These animals maintain viral persistence within their populations, facilitating recurrence. Ticks, particularly species of the *Ixodes* genus, function as vectors, acquiring the virus during blood meals from infected reservoir hosts. Subsequently, these infected ticks transmit the virus to humans and other mammals through bites, initiating viral replication and disease manifestations. Human POWV infections display a spectrum of clinical outcomes ranging from asymptomatic infections to severe neurological disorders, mainly dependent upon various host and viral factors [3].

The POWV genome consists of a positive-sense, single-stranded RNA, approximately 11,000 nucleotides in length [1]. This RNA genome encodes three structural proteins: capsid, membrane, and envelope, essential for viral assembly and entry into host cells. In addition, the genome encodes seven nonstructural proteins (NS1, NS2A, NS2B, NS3, NS4A, NS4B, and NS5), each serving vital functions in the viral life cycle. NS1 and NS2A are involved in immune evasion, virion assembly, and viral replication, while NS2B is a cofactor for NS3. NS3 functions as a protease and helicase, crucial for polyprotein processing and RNA unwinding during replication. NS5, the RNA-dependent RNA polymerase, is vital to viral RNA synthesis, and NS4A and NS4B contribute to the formation of the viral replication complex [4]. These nonstructural proteins facilitate efficient viral replication and immune modulation and represent essential molecular targets for developing antiviral therapies, as disrupting their functions could inhibit viral propagation and pathogenesis within the host. Understanding the transmission cycle components of POWV is critical for developing effective disease prevention and control strategies, as the involvement of both reservoir hosts and vector species complicates efforts to interrupt viral spread.

This review comprehensively explores the full range of neurological effects associated with POWV infections beyond the well-documented encephalitis. It delves into the molecular pathogenesis of POWV, detailing how it evades host immune defenses and the critical roles played by its nonstructural proteins.

## Review

Neurological symptoms of POWV

Encephalitis and Meningitis

Despite their rarity, POWV infections can result in severe neurological sequelae, primarily through the development of encephalitis. Yu et al. reported that encephalitis associated with POWV can lead to long-term neurological deficits or fatality, underscoring the infection's severity despite its low incidence [5]. The infrequency of transmission is mainly attributable to the virus's reliance on *Ixodes* ticks as vectors, which have lower human feeding rates than other arthropods. POWV's geographic distribution is confined to areas where both the virus and its tick vectors are endemic, primarily in northeastern and north-central North America, significantly influencing transmission rates and risk of exposure [5]. The clinical presentation of POWV encephalitis encompasses several diagnoses. Kenfak et al. noted that patients commonly present with nonspecific symptoms such as high fever, severe headaches, disorientation, and altered mental states, alongside more distinct neurological signs, including behavioral abnormalities and irritability [6]. Neurological deficits, such as limb weakness or paralysis, altered reflexes, and cranial nerve dysfunction, are frequently observed. In severe cases, POWV encephalitis can rapidly progress to life-threatening conditions, including altered consciousness, seizures, coma, and acute hypoxemic respiratory failure (AHRF), necessitating immediate medical intervention [7]. Although encephalitis is a frequent manifestation of POWV infection, meningitis is also prevalent, presenting a distinct clinical profile. Pach and colleagues described the hallmark symptoms of POWV meningitis as severe cephalalgia, cervical rigidity, photophobia, pyrexia, and generalized malaise [8]. Importantly, patients with meningitis typically retain full cognitive awareness, distinguishing it from encephalitis, which often involves cognitive dysfunction, confusion, and alterations in mental status. This distinction facilitates differential diagnosis between the two conditions.

The pathophysiological differences between meningitis and encephalitis in POWV infections are significant. Encephalitis involves inflammation of the brain parenchyma and spinal cord, leading to more severe neurological consequences and higher morbidity and mortality. In contrast, meningitis, an infection of the meninges, generally presents with more favorable outcomes due to the superficial nature of meningeal involvement and a lower likelihood of permanent neurological damage. However, untreated meningitis, particularly in POWV cases, may still progress to life-threatening complications such as cerebral edema and seizures [9]. Patients presenting with POWV encephalitis typically manifest with a rapid onset of hyperpyrexia, accompanied by severe cephalalgia, cognitive dysfunction, confusion, irritability, and notable behavioral disturbances [6,9]. Neurological manifestations involve limb paresis, abnormal deep tendon reflexes, and cranial nerve deficits, which may progress to more severe complications, including seizures, coma, and respiratory compromise [7]. Given the significant neurological involvement and potential for rapid deterioration, expedited diagnosis is critical for initiating appropriate management. The low incidence and non-specific symptoms may cause frequent initial misdiagnosis of POWV infections, particularly in regions with low prevalence rates [10].

Currently, there are no FDA-approved antiviral therapies specifically targeting POWV encephalitis or meningitis, leaving treatment options primarily supportive and symptomatic. Although ongoing research explores potential therapeutic agents, our understanding of the virus's neuropathogenic mechanisms remains incomplete. As a result, clinical management focuses on alleviating symptoms, ensuring patient comfort, and minimizing the risk of secondary infections and neurological sequelae [11]. In severe cases, hospitalization and intensive medical intervention may be required, including the administration of intravenous fluids, anticonvulsants, corticosteroids, and broad-spectrum antivirals or antibiotics to mitigate inflammation and prevent further complications [12]. Supportive measures such as antipyretics and anticonvulsants are also employed to control pyrexia and seizures while maintaining fluid balance through intravenous hydration. Early diagnosis and initiation of treatment are paramount to optimizing patient outcomes and mitigating long-term neurological consequences.

Nevertheless, even with aggressive supportive care, severe cases of encephalitis can result in permanent neurological or cognitive impairments, the severity of which may vary across individuals [12]. Thy et al. explored the prognostic disparities between viral meningitis and encephalitis, primarily focusing on POWV infection [13]. The researchers observed that patients presenting with viral meningitis, including those with POWV etiology, generally exhibited a more favorable clinical trajectory than their counterparts with encephalitis. Most viral meningitis cases were characterized by a self-limiting course, with symptomatic resolution typically occurring within a few weeks post-onset. Notably, the incidence of persistent neurological sequelae was significantly lower in this cohort. Nevertheless, the authors emphasized the critical importance of prompt medical intervention and vigilant monitoring, as these measures are instrumental in mitigating potential long-term complications.

Nervous system disorders attributable to POWV

**Figure 1:**
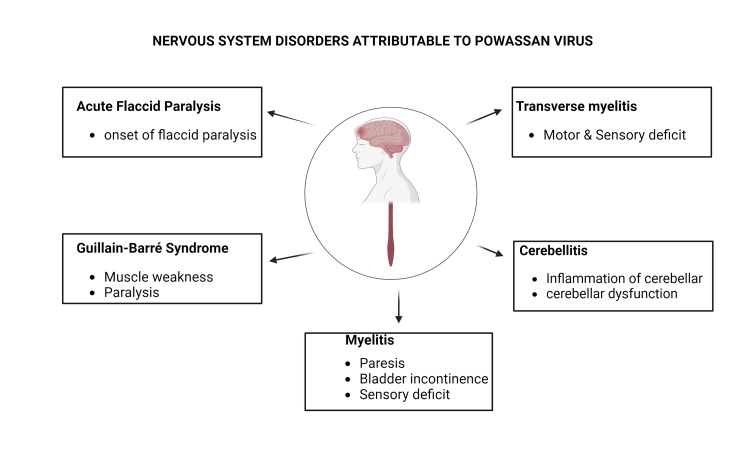
Nervous system disorders attributed to the Powassan virus The authors generated the figure using Biorender.com.

POWV has been associated with several central and peripheral nervous system disorders, including myelitis, transverse myelitis, acute flaccid paralysis (AFP), Guillain-Barré syndrome (GBS), and cerebellitis (Figure 1). These conditions exhibit distinct pathophysiological mechanisms and neuroanatomical targets, differentiating them from the more commonly observed encephalitis and meningitis. In contrast, myelitis and transverse myelitis affect the spinal cord, leading to motor and sensory dysfunctions in the extremities [14]. On the other hand, AFP and GBS are peripherally mediated neuropathies, manifesting as progressive muscle weakness and paralysis, thus diverging from the central nervous system (CNS) involvement seen in encephalitis and meningitis. Cerebellitis, a less common manifestation of POWV infection, targets the cerebellum, resulting in ataxia and developmental coordination disorder (DCD) in children [15].

AFP

AFP is characterized by the sudden onset of flaccid paralysis. Although rare, AFP represents a crucial clinical entity with diverse etiologies, including viral infections. Recent epidemiological data suggest a potential association between POWV infections and AFP cases, warranting further investigation [16]. It is essential to note that AFP can be attributed to various viral agents, including flaviviruses and enteroviruses. The pathophysiology of POWV-induced AFP remains to be fully elucidated; however, it is hypothesized that POWV exerts deleterious effects on the peripheral nervous system, particularly targeting motor neurons responsible for muscular innervation [17]. Viral-mediated damage to these motor neurons results in the characteristic muscular weakness and paralysis observed in AFP. While poliovirus, an enterovirus causing poliomyelitis, remains a predominant etiological agent for AFP, other flaviviruses have also been implicated. This viral tropism and subsequent neuronal injury disrupt neuromuscular signaling pathways, ultimately manifesting as AFP-associated muscular weakness and paralysis [18].

A comprehensive clinical assessment is needed for the definitive diagnosis of POWV-induced AFP. This evaluation necessitates a multifaceted approach, encompassing a detailed patient history, thorough physical examination, and extensive laboratory investigations. In cases of suspected POWV infection, spinal MRI may reveal characteristic abnormalities or lesions indicative of spinal cord involvement, such as inflammation or structural alterations, which can suggest the viral infection [17,18]. Electromyography (EMG) is essential in the diagnostic algorithm, quantitatively assessing muscle electrical activity and neuromuscular junction function. In POWV-induced AFP, EMG findings may demonstrate specific patterns of neurogenic injury or myopathic changes, such as chronic denervation or fibrillation potentials (positive sharp wave), correlating with the clinical presentation of muscle weakness and paralysis [19]. The efficacy of therapeutic intervention post-diagnosis significantly influences the prognostic outlook and risk of permanent neurological deficits in AFP treatments. Since management strategies primarily focus on supportive care and symptomatic relief, it is essential to note that the extent of recovery and potential for complete neurological restoration vary among persons based on the intensity and duration of physical therapy, pain management efficacy, and respiratory support requirements.

GBS

GBS, a peripheral nervous system autoimmune disorder characterized by muscle weakness and paralysis, has been hypothesized to have a link with POWV infections [20]. While GBS is more frequently associated with infections such as the Zika virus and *Campylobacter jejuni*, the postulated role of POWV in GBS pathogenesis is worth investigating. The precise immunopathological mechanisms underlying virus-induced autoimmune disorders such as GBS remain incompletely understood; however, they are believed to involve intricate immune cascades. One prominent hypothesis is molecular mimicry, wherein viral antigens exhibit structural homology with host proteins, causing an aberrant autoimmune response targeting both viral and host epitopes [21].

Additional factors contributing to an autoimmune reaction include immune dysregulation and epitope spreading. The established associations between GBS and specific pathogens, such as Zika virus and *Campylobacter jejuni*, are primarily predicated on epidemiological evidence. Based on shared pathogenic mechanisms between POWV and these viruses, researchers postulate a potential link between POWV and GBS [20]. Inflammation of the nerves and impaired nerve signal transmission are characteristic features of GBS. The clinical presentation usually initiates with weakness in the lower extremity musculature, followed by a progressive, ascending pattern of muscular debility. This characteristic progression is attributed to the autoimmune-mediated inflammation and damage to peripheral nerves. In severe cases, patients may need mechanical ventilatory support with significant respiratory muscle involvement due to phrenic nerve compromise [22]. Early diagnosis and treatment with intravenous immunoglobulin (IVIG) and plasmapheresis are crucial for improving outcomes and minimizing the risk of complications. IVIG and plasmapheresis are critical in mitigating nerve damage, expediting recovery, minimizing complications, decreasing long-term disability, and enhancing overall outcomes. These therapeutic interventions regulate the immunological response and facilitate the body's innate healing mechanisms, thus improving the likelihood of a favorable recovery [23].

Myelitis, Transverse Myelitis, and Cerebellitis

Venkatesan and Bradshaw associated the occurrence of myelitis with POWV infection, suggesting the neurotropic potential of this arbovirus to affect multiple regions within the CNS [24]. POWV-induced myelitis may present through neurological dysfunctions, including varying degrees of paresis or paralysis, autonomic disturbances such as bladder and bowel incontinence, and sensory deficits [25]. Transverse myelitis is characterized by sensory and motor deficits resulting from spinal cord inflammation, presenting as a disruption in neural signal propagation from the cerebral cortex to peripheral effectors.

Toledano elucidated an association between POWV infections and the occurrence of transverse myelitis [26]. The clinical presentation of transverse myelitis includes paresis or paralysis of the extremities, paresthesia or hypoesthesia, and urinary incontinence. The symptomatic profile in affected individuals is contingent upon the area of localized inflammation within the spinal cord. For instance, cervical spine involvement may cause upper extremity dysfunction, whereas lumbar inflammation may manifest as lower extremity impairment. The variability in symptoms reported in cases of transverse myelitis can be attributed to both the individual's distinct immunological response and the severity of inflammation [26].

Dumic et al. emphasized the necessity of conducting a clinical assessment alongside an MRI to diagnose POWV-induced transverse myelitis definitively [27]. The immune system's reaction to the POWV frequently causes inflammation within the spinal cord to be visualized. MRI scans can detect these regions of atypical signal intensity or lesions inside the spinal cord, thereby providing visual confirmation of inflammatory processes. MRI facilitates differential diagnosis as it helps distinguish transverse myelitis from other spinal cord-related conditions. Serial MRI scans are often employed to monitor disease progression and evaluate treatment effectiveness, providing insights into the course of the illness.

Cerebellitis, characterized by the inflammation of the cerebellum, a critical neural substrate for motor coordination and balance, remains a rare but documented consequence of infection by the POWV. The inflammation within the cerebellar parenchyma can disrupt standard physiological mechanisms, portraying symptoms of cerebellar dysfunction. These manifestations may include ataxia, tremors, and dysarthria [28]. Diagnosis requires a comprehensive clinical evaluation, neuroimaging, and the systematic exclusion of other possible causes of cerebellar dysfunction. Therapeutic approaches primarily focus on immunomodulation to attenuate the inflammation, with adjunctive supportive measures to mitigate cerebellar edema and preserve neuronal integrity [29].

Risk factors and transmission of POWV

**Figure 2:**
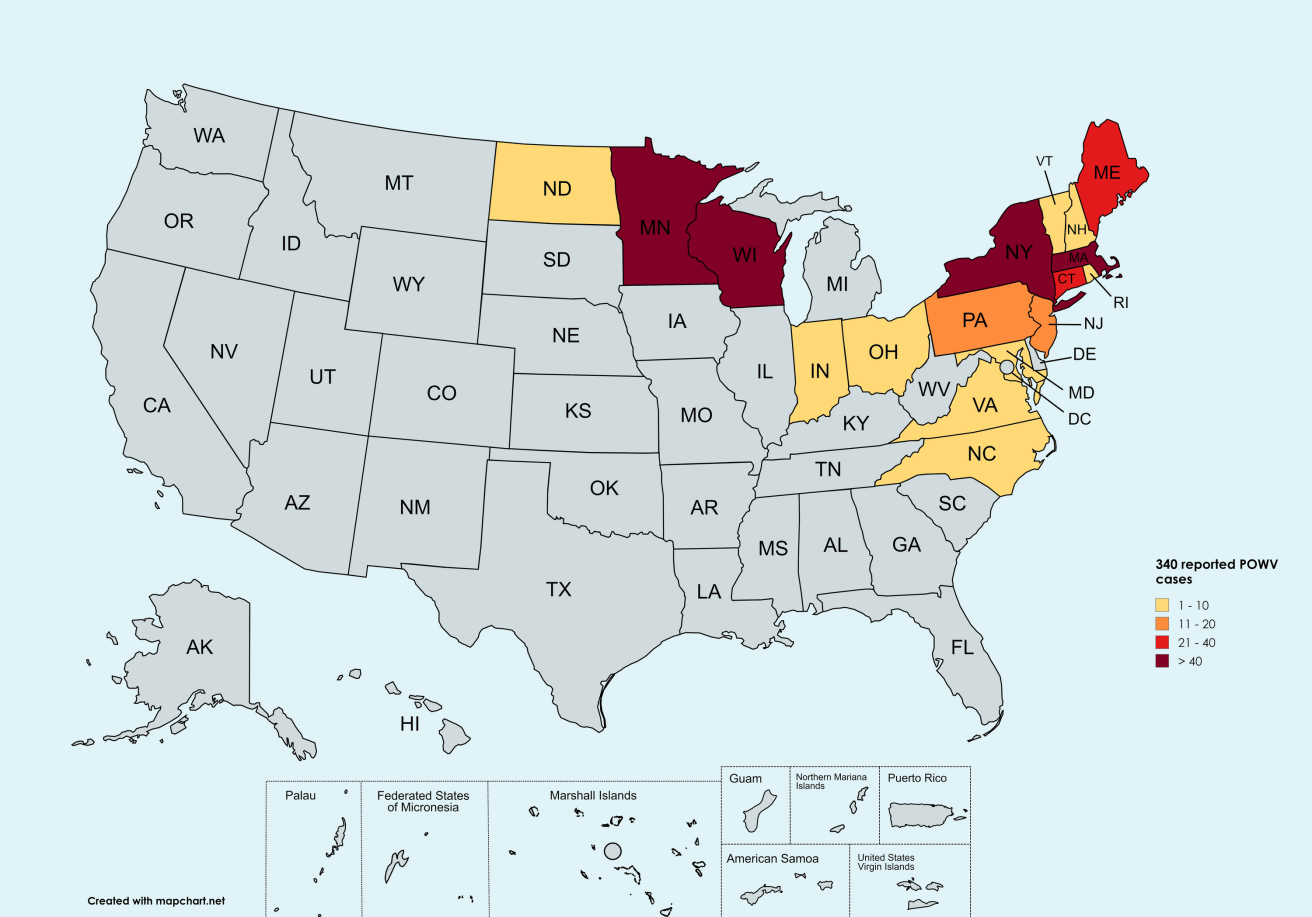
Geographical distribution of reported human cases of Powassan virus by state of residence Per CDC data report 2003-2004, Powassan virus neuroinvasive disease cases have been reported in Maine (25), Connecticut (24), Indiana (1), Maryland (1), Ohio (1), Vermont (2), Massachusetts (60), Minnesota (65), New Hampshire (9), New Jersey (15), New York (56), North Carolina (1), North Dakota (2), Pennsylvania (16), Rhode Island (6), Virginia (1), and Wisconsin (55) [36]. The authors generated the figure using mapchart.net.

Epidemiological investigations of POWV have predominantly focused on determining its prevalence and geographical distribution (Figure 2), with particular emphasis on regions with high primary tick vectors [30], *Ixodes scapularis* and *Ixodes cookei*, and their mammalian reservoir hosts [31]. The virus is endemic in North America's northeastern and north-central regions, including parts of the United States (e.g., Minnesota, Wisconsin) and Canada (e.g., Ontario, Manitoba) [31]. Furthermore, sporadic cases have been reported in European countries, including Russia, Norway, Sweden, and France, suggesting a potential expansion of its geographical range. Rupani et al. posited that the accurate diagnosis and reporting of POWV infections are often confounded by the virus's relative rarity and its symptomatic similarity to other tick-borne illnesses, potentially leading to underreporting and complicating ongoing epidemiological surveillance efforts [32]. Risk factor analysis has revealed significant associations between POWV infections and various environmental and behavioral variables. Outdoor activities such as camping, hiking, and hunting have been correlated with an increased risk of exposure to tick habitats and subsequent POWV infections. Additionally, proximity to tick-infested areas and domestic animals within households have been identified as potential risk factors [33]. Occupational considerations have also been implicated, with individuals engaged in forestry, landscaping, and other outdoor professions demonstrating heightened susceptibility to tick bites and an elevated risk of contracting tick-borne pathogens. While the primary mode of POWV transmission remains through the bite of an infected tick, Hart et al. emphasized the critical importance of comprehending tick population dynamics, reservoir host ecology, and transmission patterns for effectively implementing prevention and control strategies [34]. Moreover, the potential for POWV transmission through blood transfusions and organ transplantation has emerged as a novel concern, indicating the necessity for stringent blood safety protocols and comprehensive donor screening in POWV-endemic regions [35].

Serological, molecular, and imaging techniques

Accurately identifying POWV infections is contingent upon the conclusive findings obtained through laboratory testing [37]. Serological assays, including enzyme-linked immunosorbent assay (ELISA) and virus neutralization tests, detect virus-specific antibodies in patient sera, facilitating the confirmation of acute and convalescent POWV infections. Molecular diagnostic techniques, particularly reverse transcription-polymerase chain reaction (RT-PCR), help detect viral RNA in several clinical specimens, including blood, cerebrospinal fluid (CSF), and other tissue samples [38]. RT-PCR demonstrates optimal sensitivity during the acute phase of infection when viremia is at its peak, enabling early and accurate diagnosis. However, the molecular diagnosis of POWV is not without challenges, as potential cross-reactivity with genetically related flaviviruses may lead to false-positive results, necessitating careful interpretation of diagnostic outcomes [38]. This constant enhancement of diagnostic specificity minimizes the risk of misdiagnosis and ensures precise case identification. Developing highly specific molecular assays, potentially incorporating next-generation sequencing technologies or multiplex PCR platforms capable of differentiating between closely related flaviviruses, may provide a more robust diagnostic approach.

Javaid and Saeed analyzed the cerebral symptoms associated with POWV infections using MRI and CT imaging [39]. Characteristic neuroimaging findings include multifocal areas of hyperintensity on T2-weighted and fluid-attenuated inversion recovery (FLAIR) sequences, predominantly affecting the thalami, basal ganglia, and cortical regions. Implementing advanced MRI techniques, such as diffusion-weighted imaging (DWI) and magnetic resonance spectroscopy (MRS), further enhanced the assessment of the extent of parenchymal damage and evaluated metabolic alterations in affected cerebral tissues. Integrating state-of-the-art neuroimaging modalities with clinical and laboratory parameters can significantly augment diagnostic accuracy, provide valuable prognostic information, and inform therapeutic decision-making in POWV infections [40]. However, the limited availability of advanced imaging technologies in some clinical settings is a challenge in POWV infection testing. The availability of advanced neuroimaging, particularly in endemic regions, will facilitate early detection, accurate diagnosis, and optimal management of POWV-associated neurological complications.

Pathophysiology and immune response

POWV infects leukocytes to traverse the blood-brain barrier (BBB), facilitating viral entry into the CNS parenchyma [40]. This leukocyte-mediated viral trafficking involves interactions between viral proteins, cellular adhesion molecules, and BBB endothelial cells, ultimately compromising BBB integrity. Upon CNS penetration, POWV is hypothesized to trigger a robust neuroinflammatory response that activates resident immune cells, including microglia and astrocytes [41]. This neuroimmune activation leads to the downstream activation of pro-inflammatory cytokines, chemokines, and neurotoxic mediators, potentially exacerbating neuronal injury and contributing to the observed neurological damage. The ensuing neuroinflammation further disrupts synaptic function, alters neurotransmitter signaling, and induces oxidative stress, culminating in progressive neuronal damage. The balance between protective immunity and immunopathology is central to disease outcomes.

A robust and well-regulated immune response is also crucial for viral clearance and host recovery [42]. Alternatively, an excessive or dysregulated immune response, particularly within the CNS, can give rise to immunopathology and tissue damage. Also, gaining a deeper understanding of the intricate interplay between the innate and adaptive immune responses to POWV infections can offer novel perspectives on the evolution of illness and guide the advancement of immunomodulatory therapeutics. Importantly, characterization of the spatiotemporal dynamics of immune cell infiltration, activation states, and effector functions in POWV-infected tissues may reveal key cellular mediators of both protective immunity and immunopathology [42].

Treatment and challenges

The therapeutic landscape for POWV infections remains challenging due to the current absence of FDA-approved antiviral agents targeting this pathogen [43]. Clinical management primarily focuses on supportive care. Patients exhibiting severe meningeal symptoms typically necessitate analgesic interventions to manage headaches, alongside antiemetic treatments and rehydration strategies to address accompanying nausea and vomiting [44]. However, ongoing research efforts have focused on evaluating the efficacy of various antiviral compounds through preclinical studies and animal models. These investigations have yielded drug candidates with the potential to attenuate viral replication and mitigate disease severity. Of particular interest is exploring antiviral agents currently employed in managing other flaviviral infections, such as Zika and West Nile, for their potential therapeutic application in POWV infections [42]. The development of targeted antiviral therapies remains a research priority against POWV infections. Supportive care therapies include antipyretic measures, anticonvulsant therapy for seizure prophylaxis and management, and judicious fluid resuscitation to maintain hemodynamic stability and optimize cerebral perfusion [44]. Symptomatic management extends to addressing neurological deficits, with interventions tailored to mitigate specific impairments and improve patient comfort. Neurorehabilitation and physiotherapy facilitate functional recovery and promote autonomy in patients with POWV-induced neurological dysfunctions [45]. The implementation of individualized rehabilitation protocols has the potential to significantly improve long-term functional outcomes and health-related quality of life metrics. Moreover, integrating advanced neuroimaging modalities and neurophysiological monitoring techniques may facilitate personalized treatment approaches and enable real-time assessment of therapeutic efficacy.

**Figure 3:**
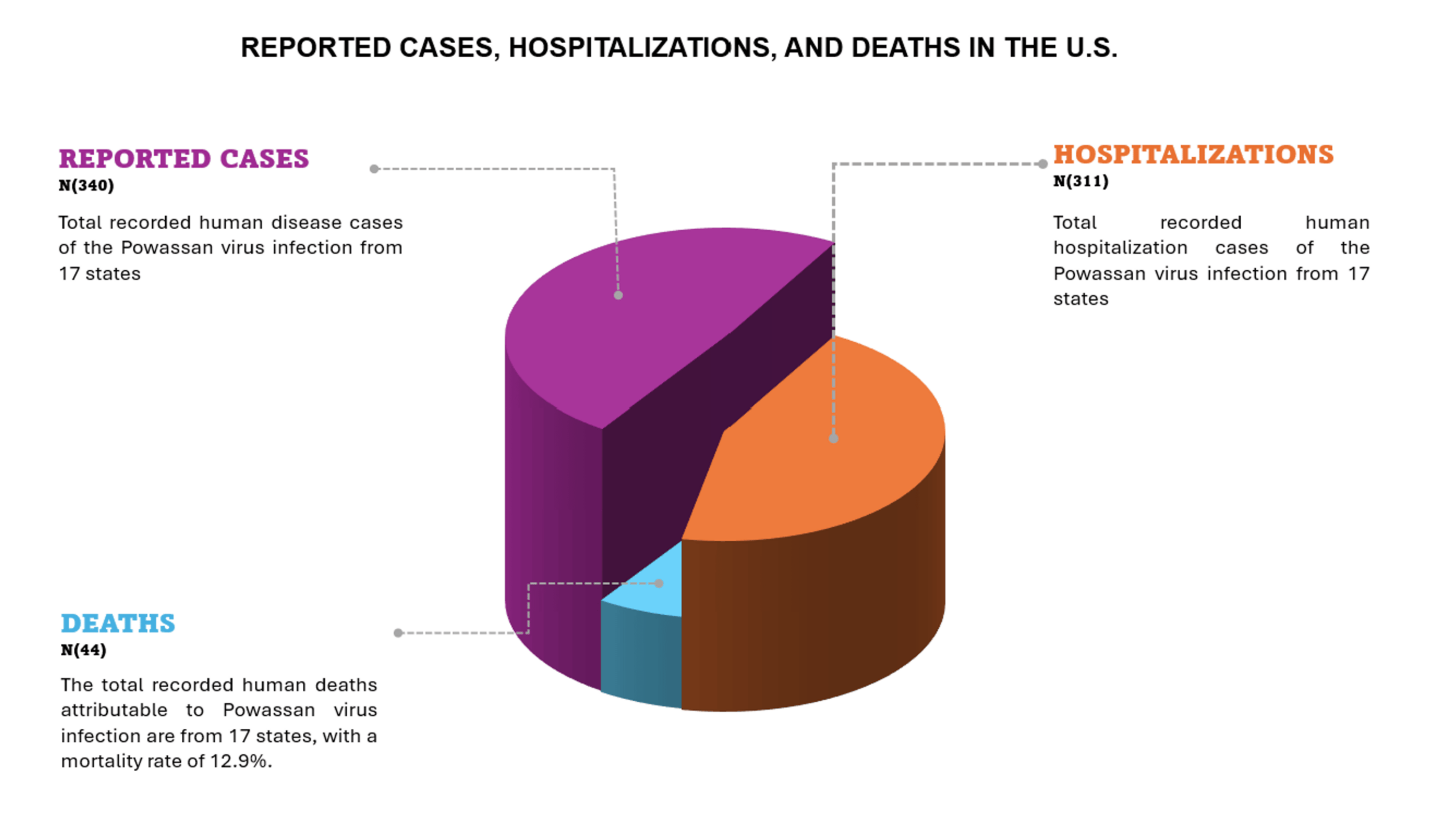
The number of human disease cases reported in the United States, associated hospitalizations, and deaths from 2004 to 2023 The figure was generated by the authors using Microsoft Office PowerPoint (Microsoft Corporation, Redmond, Washington). Data source: CDC [36].

While several antiviral compounds have demonstrated promising in vitro efficacy against POWV, their translation to in vivo models and clinical settings remains unpublished. Some research challenges include the complexities of viral tropism, BBB penetration, and the narrow therapeutic window inherent to acute viral encephalitides. The potential repurposing of antiviral agents with established efficacy against other flaviviruses, such as Zika and West Nile viruses, presents an intriguing avenue for investigation [46]. Moreover, the relatively low incidence of POWV infections (Figure 3) poses significant hurdles for conducting adequately designed clinical trials. Furthermore, innovative clinical trial designs, such as adaptive platform trials or basket trials incorporating multiple tick-borne encephalitides, may be necessary to expedite the evaluation of potential therapeutic agents that are efficient against POWV.

Gaps in the current literature

Inadequate clinical sample size is a key methodological constraint in existing POWV research. The scarcity of POWV cases and the complexities inherent in its diagnostic process present challenges in patient recruitment for in-depth investigations [47]. Consequently, there is a need to augment the statistical power of specific research findings to establish their validity and reliability. Giménez-Richarte et al. indicate that investigations confined to specific geographic regions or healthcare settings may be susceptible to selection bias, potentially compromising the generalizability of findings to broader populations [48]. Similarly, the predominant inclusion of severe cases in hospital-based studies may skew the data toward more acute manifestations of POWV infection, potentially overlooking milder presentations observed in outpatient settings. To address these limitations, research paradigms can utilize population-based data as the primary focus of investigation. Additionally, it is imperative to strategically enroll patients from inpatient and outpatient settings to mitigate potential biases and selection issues [48]. Dumic et al. have elucidated that retrospective analyses have been the predominant investigative approach in POWV infection studies, entailing extensive scrutiny of medical records and historical data [27]. While these analyses offer potential for valuable insights, they are inherently susceptible to methodological limitations, including data collection inconsistencies, information gaps, and recall bias. Furthermore, initiating longitudinal cohort studies would help study the disease trajectories and treatment outcomes more precisely. The ephemeral nature of viral infections and the logistical challenges inherent in maintaining prolonged patient surveillance present obstacles to conducting such longitudinal investigations [49]. Nevertheless, establishing enduring patient registries while actively seeking collaboration with neurologists and rehabilitation specialists will help accurately assess the long-term outcomes and quality of life experienced by survivors.

Accurately replicating human POWV infections in animal models is a persistent challenge. While animal models are crucial for researching disease pathophysiology, evaluating potential treatments, and understanding immune responses, the limited availability of suitable models obscures the advancement of the POWV infection and replication mechanisms. Carpio and Barrett emphasized the importance of directing research efforts toward improving existing animal models and developing novel ones that closely simulate human POWV infections [50]. The specific mechanisms by which the virus predominantly affects the CNS and evades immune destruction within neuronal cells remain unclarified. Hermance et al. recommend investigating the viral components and host receptors involved in POWV's neurotropism and tissue tropism with the proposed employment of advanced techniques such as single-cell RNA sequencing and high-resolution microscopy to study viral interactions within neuronal cells and elucidate the underlying mechanisms of viral invasion [51]. Furthermore, comparative analyses of viral genetic variability reveal a lower diversity in tick vectors than murine hosts, suggesting a more stringent viral control mechanism in the arthropod vector [52]. This differential selective pressure implies that the evolution of POWV is likely influenced by RNA interference (RNAi)-mediated diversification and selective constraints imposed by the different hosts [52]. The reduced genetic variability observed in tick populations suggests that these arthropod vectors may serve as evolutionary bottlenecks, potentially limiting the emergence of new viral variants. Conversely, the higher genetic diversity found in rodent hosts may provide a reservoir for viral adaptation and evolution. This dichotomy in viral genetic variability between arthropod vectors and vertebrate hosts requires a careful selection of model organisms to study the POWV infection.

Identifying and diagnosing POWV infections is challenging due to their symptomatic similarities to other viral and bacterial illnesses [53]. Shared symptoms such as pyrexia, cephalalgia, and myalgia with other prevalent viral infections may lead to misclassification and consequent diagnostic delays. Particularly in regions with established POWV activity, it is advantageous for healthcare practitioners to possess a clear understanding of the clinical spectrum of POWV infections and to incorporate this virus into their differential diagnoses [53]. Hart et al. further highlighted that due to the relatively infrequent occurrence and ongoing nature of POWV infection, many healthcare practitioners may lack familiarity with its clinical manifestation and the diagnostic methodologies employed for its detection [34]. Insufficient knowledge could result in missed opportunities for timely detection and appropriate treatment. Implementing focused medical conferences, webinars, and continuing medical education (CME) activities on POWV infections is recommended.

The absence of an approved antiviral drug creates a therapeutic void compounded by variable patient responses influenced by age, immunological status, and comorbidities. The question of the optimal timing for initiating antiviral therapy in cases of POWV infections remains unresolved. The effectiveness of treatment in preventing neurological issues and reducing virus replication may be compromised due to delays in its implementation [54]. The optimal timing for initiating antiviral medication could be ascertained through prospective clinical trials that adhere to rigorous treatment protocols and employ early intervention strategies [54]. Additionally, employing biomarkers can help diagnose the severity of POWV infections and assist in early therapeutic regimens. Clinical studies using large-scale randomized controlled trials have been challenging due to low incidence rates [4,55], paving the way for adaptive trials, which allow for the simultaneous evaluation of multiple interventions and efficiently identify effective treatments. Novel biomarkers, including serum neurofilament light chain (sNfL) levels, are being investigated as potential predictors of disease severity and treatment response [55].

## Conclusions

POWV represents a significant public health challenge due to its severe neurological disorders, including encephalitis and meningitis. Despite its relatively low incidence, the complexity of its transmission cycle involving ticks and rodents complicates prevention and control efforts. Current treatment options are predominantly supportive, with no specific antiviral therapies approved for POWV. Although not fully explicated, multiple ongoing studies expound on the molecular and immunological mechanisms underlying POWV infection. Additionally, the prognostic disparities between POWV-induced meningitis and encephalitis underscore the variability in clinical outcomes, influencing management strategies. Improving diagnostic accuracy would aid in better understanding short- and long-term neurological impacts and developing effective therapeutic interventions.
